# Suppression of Non-Random Fertilization by MHC Class I Antigens

**DOI:** 10.3390/ijms21228731

**Published:** 2020-11-19

**Authors:** Junki Kamiya, Woojin Kang, Keiichi Yoshida, Ryota Takagi, Seiya Kanai, Maito Hanai, Akihiro Nakamura, Mitsutoshi Yamada, Yoshitaka Miyamoto, Mami Miyado, Yoko Kuroki, Yoshiki Hayashi, Akihiro Umezawa, Natsuko Kawano, Kenji Miyado

**Affiliations:** 1 Laboratory of Regulatory Biology, Department of Life Sciences, School of Agriculture, Meiji University, Kanagawa 214-8571, Japan; cf190407@meiji.ac.jp (J.K.); ryouta.0724512@gmail.com (R.T.); nekomaru0@gmail.com (S.K.); hanamai8713@gmail.com (M.H.); 2 Department of Reproductive Biology, National Research Institute for Child Health and Development, Tokyo 157-8535, Japan; kwjbear@gmail.com (W.K.); myoshi1230@gmail.com (Y.M.); umezawa-a@ncchd.go.jp (A.U.); 3 Next-Generation Precision Medicine Research Center, Osaka International Cancer Institute, Osaka Prefectural Hospital Organization, Osaka 541-8567, Japan; keiichi.yoshida@oici.jp; 4 Department of Obstetrics and Gynecology, Keio University School of Medicine, Tokyo 160-8582, Japan; aki_nakamura@hotmail.co.jp (A.N.); mitsutoshi.yamada@gmail.com (M.Y.); 5 Department of Molecular Endocrinology, National Research Institute for Child Health and Development, Tokyo 157-8535, Japan; miyado-m@ncchd.go.jp; 6 Department of Genome Medicine, National Research Institute for Child Health and Development, Tokyo 157-8535, Japan; kuroki-y@ncchd.go.jp; 7 Life Science Center for Survival Dynamics, Tsukuba Advanced Research Alliance (TARA), University of Tsukuba, Ibaraki 305-8577, Japan; yoshikih@tara.tsukuba.ac.jp

**Keywords:** MHC class I, sex ratio, polyspermy block, non-random fertilization

## Abstract

Hermaphroditic invertebrates and plants have a self-recognition system on the cell surface of sperm and eggs, which prevents their self-fusion and enhances non-self-fusion, thereby contributing to genetic variation. However, the system of sperm–egg recognition in mammals is under debate. To address this issue, we explored the role of major histocompatibility complex class I (MHC class I, also known as histocompatibility 2-K^b^ or H2-K^b^ and H2-D^b^ in mice) antigens by analyzing *H2-K^b-/-^H2-D^b-/-^β2-microglobulin* (*β2M*)*^-/-^* triple-knockout (*T-KO*) male mice with full fertility. *T-KO* sperm exhibited an increased sperm number in the perivitelline space of wild-type (*WT*) eggs in vitro. Moreover, *T-KO* sperm showed multiple fusion with zona pellucida (ZP)-free *WT* eggs, implying that the ability of polyspermy block for sperm from *T-KO* males was weakened in *WT* eggs. When *T-KO* male mice were intercrossed with *WT* female mice, the percentage of females in progeny increased. We speculate that *WT* eggs prefer fusion with *T-KO* sperm, more specifically X-chromosome-bearing sperm (X sperm), suggesting the presence of preferential (non-random) fertilization in mammals, including humans.

## 1. Introduction

Alternative alleles are equally transmitted through fertilization to progeny, ensuring the balanced transmission of parental genetic material. Allorecognition is the unidirectional ability of an organism to distinguish non-self-cells from self-cells [1,2]. Self-incompatibility systems in plants [3] and hermaphroditic ascidians [2,4] are known to prevent inbreeding in hermaphroditic individuals. Closely related to vertebrates, hermaphrodite ascidian *Ciona intestinalis* specifically processes a self-incompatibility system to prevent self-fertilization [5]. *Ciona* is a valuable model for understanding the evolution of self–non-self-recognition. For example, *Hsp70* genes, which are related to immune recognition, are presumed to be ancestors of *MHC class I* and *II* genes [6]. In mammals, several gamete compatibility genes expressed on either the sperm or the egg have been identified as influencing the fertilization process [5]. The representative interacting pair of proteins is Izumo1 on the sperm and Juno on the egg plasma membrane [5].

**Figure 1:**
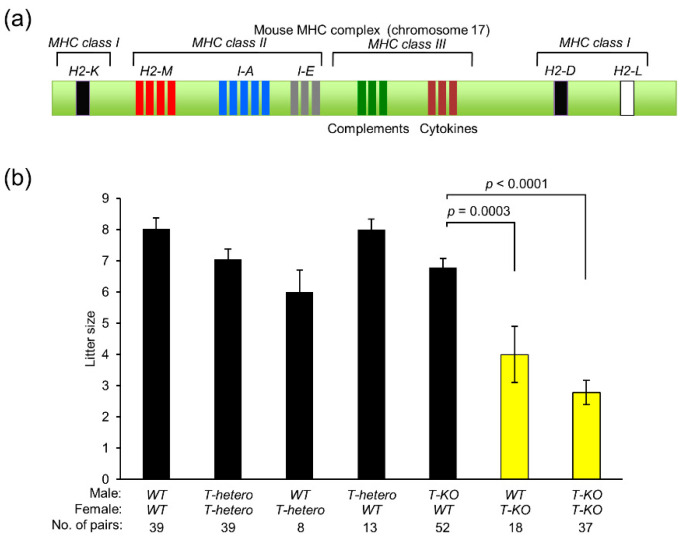
Effect of major histocompatibility complex (MHC) class I antigens on mouse fertility. (**a**) Schematic representation of MHC genes on mouse chromosome 17. The MHC gene cluster contains *MHC class I* genes (*H2-K*, *H2-D*, and *H2-L* genes) and *MHC class II* genes (*H2-M*, and *I-A* and *I-E* subregions encoding glycoproteins). Each of *H2-K*, *D*, and *L* regions contain a single gene encoding an MHC class Iα chain. Each of *I-A* and *I-E* regions contains a single gene encoding an MHC class IIα chain, and one or more genes encoding MHC class IIβ chains. The MHC class III region contains genes encoding complement proteins, Heat shock proteins, tumor necrosis factor, and lymphotoxin. The *H2-M* region contains genes encoding class IIb proteins. (**b**) Litter size of progeny obtained from intercrossing between *WT*, *T-hetero*, and *T-KO* mice. Values are expressed as mean ± SEM.

The major histocompatibility complex (MHC) mediates immune response to foreign antigens in vertebrates [7,8]. Allorecognition in vertebrates is a process by which MHC/peptide complexes, presented by donor-derived dendritic cells, are recognized directly by recipient-derived T cells [9]. MHC antigens are divided into three classical subclasses: class I, class II, and class III [10] and nonclassical molecules [11]. Mouse MHC antigens, also called histocompatibility 2 or H2 antigens, are encoded by genes located on chromosome 17 [12] (Figure 1a).

MHC class I proteins are expressed on the plasma membrane of a variety of cells. In C57BL/6 mice, the MHC class I complex consists of a membrane-spanning α-chain encoded by two MHC class I genes (*H2-D^b^* and *H2-K^b^*) and a β-chain encoded by β2-microglobulin (*β2M*) gene [13] (Figure 1a). Self- and non-self-proteins in cells are degraded in the same manner, and fragmented peptides are transported to the endoplasmic reticulum, where they bind to MHC class I antigens and are transported via the Golgi apparatus to the plasma membrane [14]. After the MHC class I complex displays antigens on the plasma membrane, cytotoxic T cells respond to non-self-antigens, leading to antibody production by B cells [14]. MHC is also known as human leukocyte antigen (HLA) in humans. HLA class I deficiency is a rare autosomal recessive immunodeficiency known as bare lymphocyte syndrome type 1 [15], which is non-life-threatening, in contrast to MHC class II deficiency, which is severe [16].

In mammalian fertilization, a single sperm fuses with an egg, because the fusion of an additional sperm is inhibited by a mechanism known as polyspermy block [5]. The polyspermy block itself comprises two mechanisms that work independently in the zona pellucida and the egg plasma membrane [5]. Sperm–egg fusion, as an immunological phenomenon, arises from allogeneic recognition between female and male gametes [2,5], implying the involvement of allogeneic MHC antigens in gamete recognition. Although several studies have reported the expression of MHC antigen of both gametes [17,18], it remains contentious.

In the present study, we explored the role of MHC class I antigens in the interaction between sperm and egg in mice.

## 2. Results

### 2.1. Contribution of MHC Class I Antigens to Mouse Fertility

To explore the contribution of MHC class I antigens to fertility, we examined litter size in mice by intercrossing between *WT*, *T-hetero*, and *T-KO* mice (Figure 1b). The litter size of *T-hetero* males and females was comparable to that of *WT* mice. The intercross between *T-KO* males and *WT* females also yielded the same number of progeny as *WT* mice. In contrast, the litter size of *T-KO* females, regardless of intercrossing with *WT* or *T-KO* males, was significantly reduced to 4.0 ± 0.9 (*p* = 0.0003) and 2.8 ± 0.4 (*p* < 0.0001), respectively, when compared to the litter size from the intercross between *T-KO* males and *WT* females (6.8 ± 0.3).

### 2.2. In Vitro Fertilization

**Figure 2:**
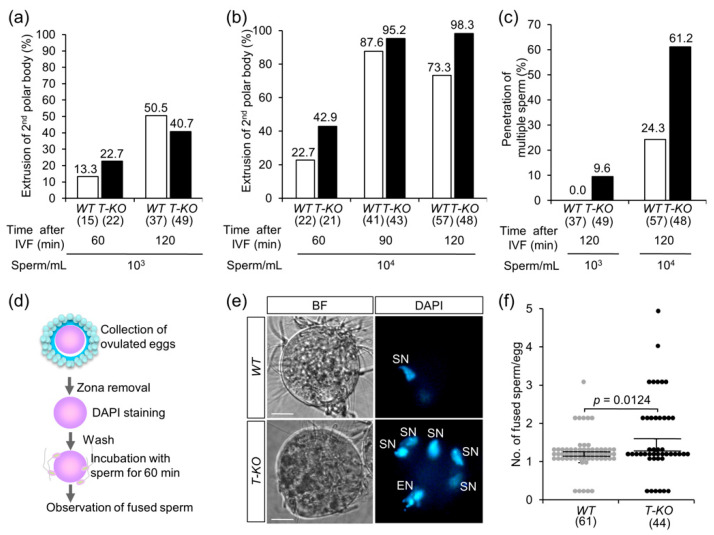
Fertilization ability of *T-KO* sperm. (**a**) The fertilization success rate (%) after ovulated eggs were incubated with *WT* or *T-KO* sperm (10^3^ sperm/mL) for 60 and 120 min. The extrusion of second polar body from the egg was designated as the success of fertilization. Parentheses indicate the number of eggs examined. (**b**) The fertilization success rate (%) after ovulated eggs were incubated with *WT* or *T-KO* sperm (10^4^ sperm/mL) for 60, 90, and 120 min. The extrusion of second polar body was designated as the success of fertilization. Parentheses indicate the number of eggs examined. (**c**) The rate of polyspermy. Eggs penetrated by multiple sperm were defined as eggs that experienced polyspermy. Ovulated eggs were incubated with *WT* or *T-KO* sperm (10^3^ sperm/mL or 10^4^ sperm/mL) for 120 min. Parentheses indicate the number of eggs examined. (**d**) Experimental flow of sperm–egg fusion assay. (**e**) Representative images of *WT* eggs fused with *T-KO* and *WT* sperm. BF: bright-field microscopy; SN: sperm nucleus; EN: egg nucleus. Scale bar: 20 µm. (**f**) The number of sperm fused with an egg. Values are expressed as mean ± SEM. Parentheses indicate the number of eggs examined.

To address the possible preferential fertilization of gametes, we performed in vitro fertilization (IVF) using *T-KO* and *WT* sperm. Eggs were isolated from the oviducts of superovulated *WT* female mice. Sperm were collected from the epididymides of *T-KO* and *WT* male mice and incubated with the eggs for 60 and 120 min. When the sperm were added to the eggs at a final concentration of 1.5 × 10^3^ sperm/mL, both sperm types fused time-dependently with the eggs (Figure 2a). The time-dependent fusion of both sperm types with eggs was observed even when a higher concentration of sperm (1.5 × 10^4^ sperm/mL) was added to eggs and incubated for 60, 90, and 120 min (Figure 2b). Polyspermy occurs when multiple sperm penetrate into the zona pellucida of an egg and are rarely fused with the plasma membrane of the egg [19,20]. Under normal physiological conditions, polyspermy is blocked at the zona pellucida and the egg plasma membrane. However, when we counted the number of sperm accumulated within the perivitelline space, an interspace between the zona pellucida and the egg plasma membrane, we found that multiple *T-KO* sperm penetrated the zona pellucida of the egg, when compared to the *WT* sperm (Figure 2c). From this result, we assumed that eggs have a low ability to block polyspermy against *T-KO* sperm in the zona pellucida.

### 2.3. Multiple Sperm Fusion

To study the polyspermy block against *T-KO* sperm, we performed a fusion assay using denuded (cumulus- and zona-free) *WT* eggs. *WT* sperm were used as control. As depicted in Figure 2d, cumulus cells and the zona pellucida were removed from ovulated eggs, and the eggs were incubated with *T-KO* and *WT* sperm for 60 min. To detect the sperm fused with the egg plasma membrane, the eggs were preloaded with DAPI. After the sperm were incubated with eggs for 60 min, the number of fused sperm per egg was counted. When *WT* sperm were added, a single sperm was fused to an egg (1.03 ± 0.06). On the other hand, when *T-KO* sperm were added, the number of sperm that fused to an egg increased significantly (1.43 ± 0.16; *p* = 0.0124) (Figure 2e,f). This result indicates that the mechanism of polyspermy block on the egg plasma membrane is weaker against *T-KO* sperm. These results suggest that the ability of the polyspermy block is reduced both at the zona pellucida and the egg plasma membrane, depending on the sperm genotype.

### 2.4. Progeny from T-KO Males and WT Females

**Figure 3:**
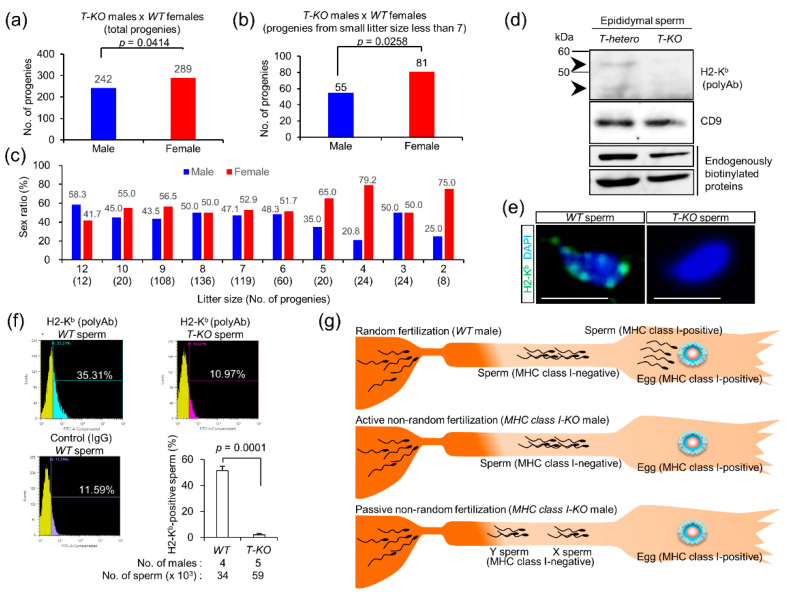
MHC class I antigens expressed on the sperm and progeny of *MHC class I* homozygous male and *WT* female. (**a**) Sex ratio in progeny of *T-KO* males and *WT* females (total neonates). (**b**) Sex ratio in progeny of *T-KO* males and *WT* females (litter size from 2 to 6). (**c**) Sex ratio in progeny dependent on the litter size. The values were analyzed by a chi-squared test. Values in parentheses indicate the number of neonates. (**d**) Immunoblotting with anti-H2-K^b^ polyAb in sperm extracts from *T-hetero* and *T-KO* males. CD9 and endogenously biotinylated proteins were detected as an internal control. (**e**) Immunocytochemical analysis of *WT* and *T-KO* sperm. The sperm were treated with anti-H2-K^b^ polyAb, and their nuclei were counterstained with DAPI. Scale bar: 10 µm. (**f**) Flow cytometric analysis of H2-K^b^ in the *WT* sperm. As negative controls, *T-KO* sperm were treated with anti-H2-K^b^ polyAb and *WT* sperm were treated with preimmune IgG. In histograms, the percentage of H2-K^b^-positive sperm was determined by calculating the sperm number shifted to the right side, relative to the peak of *WT* sperm treated with preimmune IgG. (**g**) Hypothesis of male-biased fertilization. We assume that the sperm population from *WT* males could be divided into two groups: MHC-class-I-positive and MHC-class-I-negative population. MHC-class-I-negative population would be divided into two groups: X chromosome-bearing sperm (X sperm) and Y chromosome-bearing sperm (Y sperm). Under normal physiological conditions, MHC-class-I-committed random fertilization occurs, but MHC-class-I-negative sperm are not involved in fertilization. When the MHC-class-I-positive sperm population disappears, MHC-class-I-negative sperm, more specifically the X sperm, fertilizes MHC-class-I-positive eggs.

To address this issue, we intercrossed *T-KO* males with *WT* females. When the sex ratio was examined in the progeny, there was no significant difference in the proportion of males to that of females (Figure 3a). Internal and external factors, such as oxygen stress and exposure to high temperature, have been reported to render the proportion of males smaller than females in the litter [21]. Thus, when progeny born in the small litter (average of less than 7) were examined, the proportion of females increased significantly (Figure 3b). Furthermore, the proportion of females and males was changed sequentially from small to large litter sizes (Figure 3c). When the litter size was less than the average value of 7, the proportion of females increased.

### 2.5. Expression of MHC Class I Antigens on Sperm

Previous studies [17] have reported the expression of MHC class I antigens in human sperm, but its expression in mice [18] is still controversial. Hence, we examined the expression of MHC class I antigens in mouse sperm by immunoblotting and flow cytometry. We used two commercially available antibodies: rabbit anti-H2-K^b^ polyclonal antibody (polyAb) and mouse anti-H2-K^b^/H2-D^b^ monoclonal antibody (mAb). CD9, which is a major component of exosomes, is expressed on mouse eggs as an essential part of fusion-promoting microexosomes [22,23,24] and is highly expressed in mouse sperm [25]. In addition, two major endogenously biotinylated proteins are expressed in various tissues and sperm in mice [26,27]. We detected these proteins as internal controls.

To examine alterations in the quantities of MHC class I antigens in mice, sperm were collected from the epididymides of *T-hetero* and *T-KO* male mice. As shown in Figure S1, mouse anti-H2-K^b^/H2-D^b^ mAb did not interact with the mouse sperm extracts, because it cross-reacted with the endogenous mouse immunoglobulin G (IgG). Instead, rabbit anti-H2-K^b^ polyAb immunoreacted with 45 kDa and 65 kDa proteins in the sperm extracts of *T-hetero* males, but not with the *T-KO* sperm (Figure 3d and Figure S2).

To examine the cell surface expression of MHC class I antigens on sperm, *WT* sperm were subjected to immunocytochemical and flow cytometric analyses. We used *T-KO* sperm immunostained with anti-H2-K^b^ polyAb and *WT* sperm incubated with preimmune IgG as negative controls. *WT* sperm immunoreacted with this antibody, and the patched pattern was observed on the sperm head (Figure 3e and Figure S3). When flow cytometric analysis was performed, immunostained *WT* sperm were limited to 23.72%, compared to *WT* sperm treated with preimmune IgG. Similarly, immunostained *WT* sperm were limited to 24.34%, compared to the *T-KO* sperm treated with anti-H2-K^b^ polyAb (Figure 3f). This result indicates that the sperm expressing MHC class I antigens are small in number.

### 2.6. Possible Occurrence of Non-Random Fertilization

We summarized the abovementioned results in Figure 3g. MHC class I homozygosity in males with reduced fertility conditions caused the proportion of females to increase (Figure 3a–c). MHC class I antigens were expressed in a restricted population of the *WT* sperm (Figure 3f).

Under normal physiological conditions, in the presence of MHC-class-I-positive sperm, MHC-class-I-positive eggs are fertilized, and progeny with a normal sex ratio (upper image in Figure 3g) is produced. On the contrary, when MHC-class-I-positive sperm disappear, MHC-class-I-positive eggs are fertilized by MHC-class-I-negative sperm (middle image in Figure 3g). If non-random fertilization occurs actively, MHC-class-I-positive eggs prefer to fuse with MHC-class-I-negative X chromosome-bearing sperm (X sperm), as compared to Y chromosome-bearing sperm (Y sperm).

On the contrary, age-dependent physiological changes, such as oxidative stress, high temperature, and low pH, exclusively retard the motility of Y sperm [21]. Under such conditions, X sperm would be normally motile and arrive earlier at the eggs than Y sperm, presumably leading to an increase in the female proportion in the progeny by passive non-random fertilization (lower image in Figure 3g). Whether MHC-class-I-positive eggs prefer fusion with MHC-class-I-negative X sperm is unknown, but the nature of the stress-sensitive Y chromosome is predicted to largely contribute to the difference between X and Y sperm.

**Figure 4:**
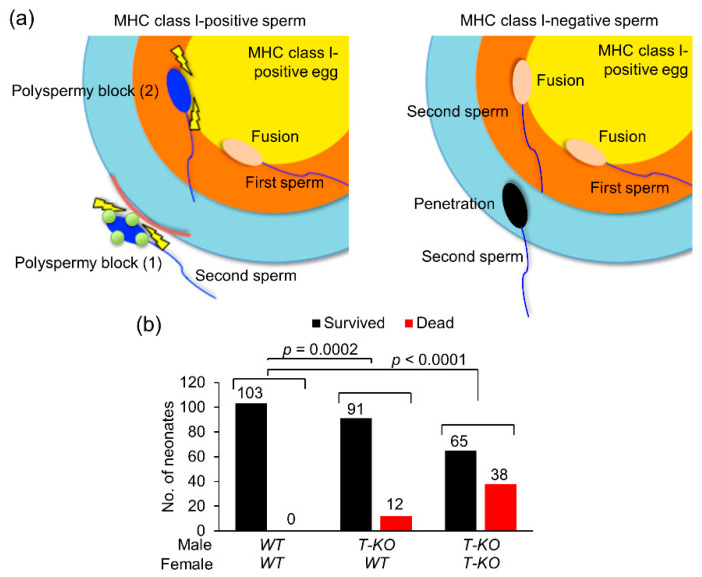
Schematic model of the role of MHC class I antigens in fertilization. (**a**) Fertilization by *WT* sperm. In the final process of fertilization, a sperm penetrates the zona pellucida and reaches the egg plasma membrane. After the sperm fuses with the *WT* egg plasma membrane, polyspermy block (1), a mechanism of polyspermy block is evoked, whereby extracellular release of egg materials is modified, which then blocks penetration by other sperm. Moreover, even if a second sperm arrives at the egg plasma membrane, polyspermy block (2), a second mechanism of polyspermy block occurs, due to which the sperm is unable to fuse with the egg plasma membrane. On the other hand, in fertilization with *T-KO* sperm, both mechanisms of polyspermy block are weak, and multiple sperm can penetrate and fuse with the *WT* egg plasma membrane. (**b**) Neonatal death. The number of dead neonates and the ones that survived was compared among three mating combinations: *T-KO* male and *WT* female, *T-KO* male and *T-KO* female, and *WT* male and *WT* female. The values were analyzed by a chi-squared test.

Taken together, our results suggest that, although non-random fertilization occurs in vivo (Figure 3) and is preferred in vitro (Figure 2 and Figure 4a), MHC-class-I-mediated mechanisms suppress non-random fertilization under normal physiological conditions both in vivo and in vitro and ensure random fertilization. In mice, soon after birth, neonates with any health problems often die, or their mothers eat them. Upon non-random fertilization, the sperm genome carrying life-threatening mutations could be transmitted to the progeny via *WT* eggs, leading to an increase in neonatal death.

To address this issue, we examined the incidence of neonatal death 24 h after birth in mice. The number of dead neonates from two types of mating pairs, *T-KO* male and *WT* female, and *T-KO* male and *T-KO* female mice, were compared with those of *WT* males and *WT* females (Figure 4b). Since the litter size of *T-KO* males and *T-KO* females was small (Figure 1b), neonatal death was expected to increase. As expected, the number of dead neonates of *T-KO* males and *T-KO* females was enhanced. The number of dead neonates was low for *T-KO* males and *WT* females, compared to that of *T-KO* males and *T-KO* females, but was significantly higher than that of *WT* males and *WT* females (Figure 4b), implying that health problems tend to occur in neonates of not only *T-KO* males and *T-KO* females, but also *T-KO* males and *WT* females.

## 3. Discussion

In populations with high genetic variations, random fertilization increases genetic diversity. In contrast, since random fertilization is unable to rapidly create new genetic variations, the genetic diversity is reduced in populations with low genetic variations [28]. Instead, when non-random fertilization occurs preferentially between sperm and egg carrying genetically distinct genomes, it actively creates new genetic variations and enhances genetic diversity, even in populations with low genetic variations. In the present study, MHC-class-I-mediated mechanism strongly suppressed non-random fertilization in vivo, ensuring random fertilization.

### 3.1. Sperm–Egg Compatibility

Mammalian fertilization has two steps to prevent polyspermy at the egg plasma membrane and zona pellucida (Figure 4a). If the initiation of polyspermy block depends on sperm genotype, the efficiency of sperm fusion with the egg is largely affected, as shown in Figure 2e,f and Figure 4a. Sperm–egg fusion shares a common nature with virus–cell fusion [29]. The locus controlling persistent viral infection was mapped to the *H2-D* region of MHC class I [30]. Mutations in the *H2-D* gene reduce or delay virus clearance [31]. However, the relationship between MHC class I antigens, sperm–egg fusion and polyspermy block remains unclear.

MHC class I antigens are expressed on a variety of cells, and their expression has been reported in human sperm [32]. In contrast, both sperm and eggs have been reported to lack MHC class I antigens in previous studies [33], implying that the expression of MHC class I antigens may be at a minimum detectable level. It has also been reported that soluble MHC class I is released from grafted tissues after organ transplantation [34], implying its contribution to immune responses against grafted tissues. Correspondingly, the dot-like localization of MHC class I antigens was detected on the sperm (Figure 3e). Therefore, secreted forms of MHC class I antigens released from the sperm could be involved in sperm–egg compatibility.

### 3.2. Sperm Heterogeneity

Generally, since intercellular bridges connect spermatocytes until the end of spermatogenesis, cellular materials, including proteins, are separated equally between mature epididymal sperm, regardless of the genotype (*WT*, heterozygote, or homozygote) [35]. Correspondingly, recent studies have denied morphological differences between X and Y sperm [26]. As shown in this study, MHC class I antigens were expressed on sperm, but MHC-class-I-positive sperm were limited to around 24% (Figure 3d–f) of the total population, implying that its surface localization might be restricted. Similarly, the distribution of cell surface proteins is known to be heterogeneous in sperm [36].

Many studies have reported the heterogeneity of sperm morphology and behaviors in human sperm, and seasonal morphological variations have been observed in boar sperm [37]. There is no significant relationship between sperm heterogeneity and male fertility in most cases [37]. We assume that sperm heterogeneity maintains male fertility in mammals (Figure 3).

### 3.3. Differences between X and Y Sperm

Since the difference between the DNA content of X and Y sperm is evident, recent studies have focused on their proteomic differences, depending on sex chromosomes [26], and raising the possibility that their physiological differences may emerge under some conditions, presumably inside the female reproductive tract. Previous studies on aneuploidy also provide evidence of the selective removal of Y sperm in mice and humans, leading to enhanced X sperm transmission [21]. Sperm motility is affected by intrauterine environmental changes, which are caused by oxidative stress, high temperature, and low pH. These factors selectively retard the motility of Y sperm [21].

The fragility of Y sperm has been discussed in terms of high mutuality in human and mouse Y chromosomes because of its unique genomic structure, when compared to other chromosomes [38]. In particular, human and mouse Y chromosomes do not recombine with a homologous X chromosome, but they contain large segments arranged in palindromes that often bend and cause self-recombination [39].

Studies on sperm viability show that X sperm survives better under unusual conditions such as high temperature or exposure to an endocrine disruptor, when compared to Y sperm, due to its higher DNA content [21]. However, the activation of Toll-like receptor 7/8 (TLR7/8) protein decreases the motility of X sperm [40].

### 3.4. Random Fertilization vs. Non-Random Fertilization

As mentioned above, non-random fertilization actively creates new genetic variations and enhances genetic diversity [28]. Conversely, when the transmission balance of sex ratio and genomes is important, random fertilization is useful, regardless of genomic diversity. For example, if non-random fertilization occurs, the sperm genome carrying life-threatening mutations could be transmitted from mutant sperm to progeny via *WT* eggs (Figure 4b). Genotype-independent and random segregation of sperm and eggs at fertilization is the foundation of heredity. Non-random fertilization potentially occurs in vitro, but it is highly restricted in vivo in laboratory mice, in an MHC-class-I-mediated manner.

As shown in Figure 1b, MHC class I antigens are also expected to play a role in female fertility. MHC class I antigens probably regulate both male and female fertility. In this study, we focused on *T-KO* sperm function at fertilization, because the causes of female fertility problems are diversified. Our present study contributes to the understanding of the molecular mechanism related to the suppression of non-random fertilization, but the cause of MHC-class-I-related female fertility problems remains unsolved.

## 4. Materials and Methods

### 4.1. Antibodies

A rabbit polyclonal antibody (polyAb) against a synthetic peptide corresponding to H2-K^b^ (ab93364) (Abcam PLC., Cambridge, MA, USA) was used for immunoblotting. Mouse anti-H2-K^b^/H2-D^b^ monoclonal antibody (mAb) (clone No. 28-8-6) (BioLegend, Inc., San Diego, CA, USA) was used for immunoblotting. Rat anti-mouse CD9 mAb (KMC8) and horseradish peroxidase (HRP)-conjugated streptavidin were purchased from BD Biosciences (San Jose, CA, USA). HRP-conjugated secondary antibodies (Sigma-Aldrich, St. Louis, MO, USA) were used for immunoblotting.

### 4.2. MHC-Class-I- and β2M-Deficient Mice

Triple-heterozygous (*T-hetero*) mouse embryos with a C57BL/6J background were purchased from the Jackson laboratory [13] and transferred to the oviducts of pseudopregnant female ICR mice. *T-hetero* females were mated with *T-hetero* males to yield *T-KO* mice progeny. The genotypes of the progeny were identified using sets of primers and standard procedures (Table S1). For in vitro fertilization (IVF), 8- to 12-week-old female and male C57BL/6J mice were purchased from Japan SLC Inc. (Shizuoka, Japan). The sex ratio was determined by observing the sexual dimorphism of external genitalia. The number of neonates who died by cannibalization and abandonment in 24 h after birth was counted.

All mice were housed under specific pathogen-free controlled conditions. Food and water were available ad libitum. The procedures for performing animal experiments were approved by the Institutional Animal Care and Use Committee of the National Research Institute for Child Health and Development (approval letter number: A2004-004).

### 4.3. Immunoblotting

Sperm were collected from the epididymis of 12- to 20-week-old *T-hetero* and *T-KO* males. The samples were lysed in Laemmli sodium dodecyl sulfate (SDS) sample buffer containing 2% SDS, 62.5 mM Tris-HCl (pH 6.8), 0.005% bromophenol blue, and 7% glycerol. The lysed samples were boiled for 10 min at 95 °C, and resolved by sodium dodecyl sulfate polyacrylamide gel electrophoresis (SDS-PAGE) using 12% acrylamide gels, before immunoblotting. The samples were then reduced with β-mercaptoethanol. Detection of the proteins and primary antibodies of interest was performed by enzyme-linked color development with HRP-conjugated secondary antibodies.

### 4.4. Flow Cytometric and Immunocytochemical Analyses

Sperm were collected from the epididymis of 12- to 20-week-old *T-KO* and *WT* males. Without fixation and permeabilization, the sperm from samples (2 × 10^6^ sperm/sample) was incubated on ice for 30 min in a blocking buffer containing 1% bovine serum albumin (BSA) and 3% fetal bovine serum (FBS) in phosphate-buffered saline (PBS), which blocked nonspecific binding of antibodies. To examine the expression of H2 antigens on the sperm surface, the sperm were incubated with anti-H2-K^b^ polyAb in the staining buffer for 1 h at 4 °C, for flow cytometry (using 1% FBS in PBS). After washing thrice with flow cytometry staining buffer, the sperm were incubated for 1 h at 4 °C with the HRP-conjugated secondary antibody. The sperm were then washed thrice and analyzed with an SH800 cell sorter (Sony Biotechnology Inc., Tokyo, Japan). The sperm immunoreacted with this antibody were stained with DAPI and observed under a fluorescent microscope (BZ-X710; KEYENCE, Osaka, Japan).

### 4.5. IVF

Eggs were collected from the oviductal ampulla of superovulated C57BL/6J females (8- to 12-week-old), 14 to 16 h after human chorionic gonadotropin (hCG) injection, and were placed in a 30 µL drop of Toyoda, Yokoyama, Hoshi (TYH) medium covered with paraffin oil (Nacalai Tesque, Inc., Kyoto, Japan), equilibrated with 5% CO_2_ in air at 37 °C. Sperm were collected from the epididymides of 8- to 12-week-old C57BL/6J (*WT*) male and *T-KO* male mice, and sperm capacitation was induced by incubation in TYH medium for 90 min at 37 °C in 5% CO_2_ before insemination. The final concentrations of sperm added to the eggs were 1.5 × 10^3^ sperm/mL and 1.5 × 10^4^ sperm/mL from *WT* male and *T-KO* male mice. To observe the phenomenon of multiple sperm penetration into the zona pellucida (polyspermy) of eggs, the eggs were stained with 4′,6-diamidino-2-phenylindole (DAPI) (WAKO Pure Chemical Industries, Ltd., Osaka, Japan) and observed under an LSM 510 confocal microscope (Zeiss, Thornwood, NY, USA) at 60, 90, and 120 min after insemination.

### 4.6. Examination of Sperm–Egg Fusion

To count the number of sperm fused per egg, zona-free eggs were prepared according to a previously described procedure [41]. They were then preloaded with DAPI at a final concentration of 5 µg/mL in TYH medium for 20 min at 37 °C and washed three times in separate drops of TYH medium before insemination. DAPI is a fluorescent dye that can slowly permeate the living cell membrane (semi-permeable) and will not leak out of cells after washing, thus enabling the staining of only fused sperm nuclei, resulting from the transfer of DAPI into sperm after membrane fusion. The final concentration of sperm added to the eggs was 1.5 × 10^4^ sperm/mL. One hour after incubation in a 30 µL drop of TYH medium, the eggs were fixed with 2-[4-(2-hydroxyethyl)piperazin-1-yl]ethanesulfonic acid (HEPES)-buffered saline containing 2% paraformaldehyde, 0.1% glutaraldehyde, and 0.1% polyvinylpyrrolidone for 30 min at 22 °C. Next, the number of sperm fused per egg was determined by counting DAPI-transferred sperm.

### 4.7. Statistical Analyses

The statistical significance of the proportion of genotypes, and the proportion of females and males was analyzed using a chi-squared test. Significant differences (*p*-values) of the percentage of H2-K^b^-positive sperm and the frequency of fusion of egg and sperm were calculated by performing a Student’s *t*-test. A value less than 0.05 (*p* < 0.05) was considered significant. Values are expressed as mean ± standard error (SEM).

## Supplementary Materials

Supplementary Materials can be found at https://www.mdpi.com/1422-0067/21/22/8731/s1. Figure S1. Immunoblotting with anti-H-2K^b^/H2-D^b^ monoclonal antibody. Figure S2. Immunoblotting with anti--H2-K^b^ (polyclonal), CD9 (monoclonal) antibodies, and horseradish peroxidase (HRP)-conjugated streptavidin. Figure S3. Immunofluorescence of H2-K^b^ in sperm.

## Abbreviations

β2M	β2-microglobulin
BF	bright-field microscopy
DAPI	4′,6-diamidino-2-phenylindole
EN	egg nucleus
FBS	fetal bovine serum
H2	histocompatibility 2
hCG	human chorionic gonadotropin
HEPES	2-[4-(2-hydroxyethyl)piperazin-1-yl]ethanesulfonic acid
HLA	human leukocyte antigen
HRP	horseradish peroxidase
IgG	immunoglobulin G
IVF	in vitro fertilization
MHC	major histocompatibility complex
mAb	monoclonal antibody
polyAb	polyclonal antibody
SDS-PAGE	sodium dodecyl sulfate-polyacrylamide gel electrophoresis
SEM	standard error of the mean
SN	sperm nucleus
T-hetero	triple-heterozygous
T-KO	triple-homozygous
TLR	toll-like receptor
TYH	Toyoda, Yokoyama, Hoshi medium
WT	wild-type
BSA	bovine serum albumin
