# Supplementary material for: Suppression of Non-Random Fertilization by MHC Class I Antigens
Source: Int J Mol Sci. 2020 Nov 19;21(22):8731. doi: 10.3390/ijms21228731 (PMC7699254; doi:10.3390/ijms21228731)
Supplement: Supplementary file 1 [file ijms-21-08731-s001.pdf]

International Journal of
Molecular Sciences

MDPI

Article

Suppression of Non-Random Fertilization by MHC Class I Antigens

Junki Kamiya [1,†], Woojin Kang [2,†], Keiichi Yoshida [3,†], Ryota Takagi [1], Seiya Kanai [1], Maito Hanai [1], Akihiro Nakamura [4], Mitsutoshi Yamada [4], Yoshitaka Miyamoto [2], Mami Miyado [5], Yoko Kuroki [6], Yoshiki Hayashi [7], Akihiro Umezawa [2], Natsuko Kawano [1,*] and Kenji Miyado [2,*]

[1] Laboratory of Regulatory Biology, Department of Life Sciences, School of Agriculture, Meiji University, Kanagawa 214-8571, Japan; cf190407@meiji.ac.jp (J.K.); ryouta.0724512@gmail.com (R.T.); nekomaru0@gmail.com (S.K.); hanamai8713@gmail.com (M.H.)
[2] Department of Reproductive Biology, National Research Institute for Child Health and Development, Tokyo 157-8535, Japan; kwjbear@gmail.com (W.K.); myoshi1230@gmail.com (Y.M.); umezawa-a@ncchd.go.jp (A.U.)
[3] Next-generation Precision Medicine Research Center, Osaka International Cancer Institute, Osaka Prefectural Hospital Organization, Osaka 541-8567, Japan; keiichi.yoshida@oici.jp
[4] Department of Obstetrics and Gynecology, Keio University School of Medicine, Tokyo 160-8582, Japan; aki_nakamura@hotmail.co.jp (A.N.); mitsutoshi.yamada@gmail.com (M.Y.)
[5] Department of Molecular Endocrinology, National Research Institute for Child Health and Development, Tokyo 157-8535, Japan; miyado-m@ncchd.go.jp
[6] Department of Genome Medicine, National Research Institute for Child Health and Development, Tokyo 157-8535, Japan; kuroki-y@ncchd.go.jp
[7] Life Science Center for Survival Dynamics, Tsukuba Advanced Research Alliance (TARA), University of Tsukuba, Ibaraki 305-8577, Japan; yoshikih@tara.tsukuba.ac.jp
* Correspondence: nkawano@meiji.ac.jp (N.K.); miyado-k@ncchd.go.jp (K.M.)
† These authors contributed equally to this work.

Received: 22 October 2020; Accepted: 17 November 2020; Published: date

Table of content

Supplemental figure 1 (S1). Immunoblotting with anti-H-2K^b/H2-D^b monoclonal antibody

Supplemental figure 2 (S2). Immunoblotting with anti--H2-K^b (polyclonal), CD9 (monoclonal) antibodies, and horseradish peroxidase (HRP)-conjugated streptavidin

Supplemental figure 3 (S3). Immunofluorescence of H2-K^b in sperm

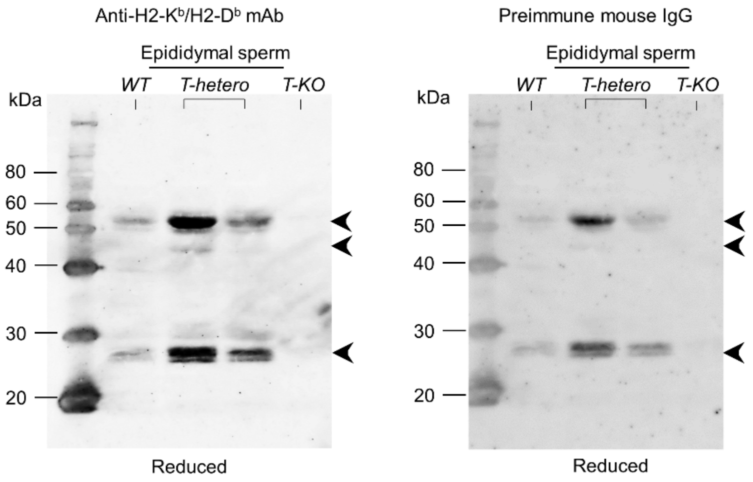

Figure S1. Immunoblotting with anti-H-2K^b/H2-D^b monoclonal antibody. mAb, monoclonal antibody; Reduced, with a disulfide-reducing agent (2-mercaptoethanol). Preimmune mouse IgG was used as a negative control for immunoblotting of H-2K^b/H2-D^b.

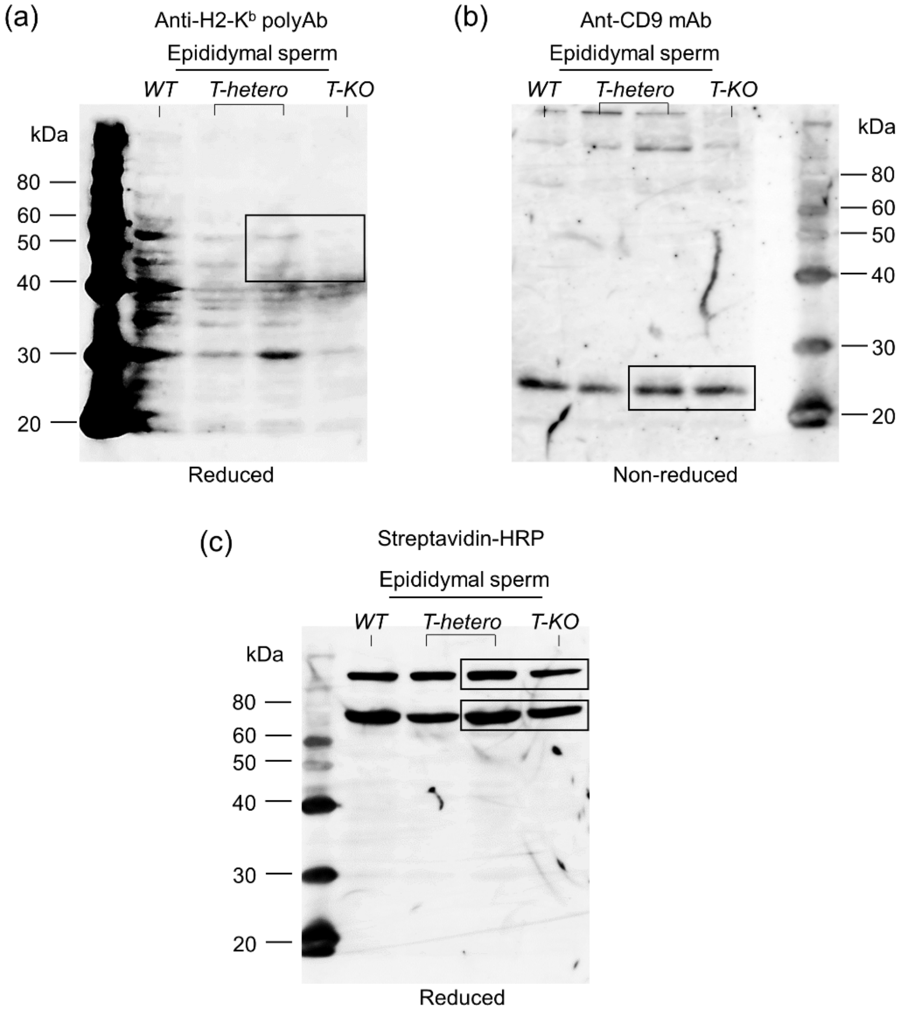

Figure S2. Immunoblotting with anti-H2-K^b (polyclonal) (**a**), CD9 (monoclonal) antibodies (**b**), and horseradish peroxidase (HRP)-conjugated streptavidin (**c**). PolyAb, polyclonal antibody; mAb, monoclonal antibody; Reduced, with a disulfide-reducing agent (2-mercaptoethanol); Non-reduced, without a disulfide-reducing agent. Boxes indicate bands represented in Figure. 3d.

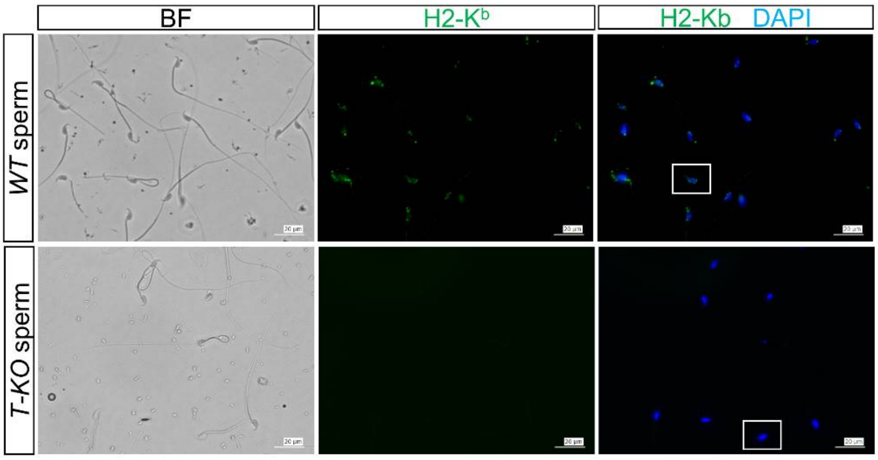

Figure S3. Immunofluorescence of H2-K^b in sperm. The sample was incubated with H2-K^b polyclonal antibody. BF, bright-field; DAPI, nucleus. Boxes indicate images represented in Figure 3e. Scale bars, 20 μm.
